# Myocardial RNA Sequencing Reveals New Potential Therapeutic Targets in Heart Failure with Preserved Ejection Fraction

**DOI:** 10.3390/biomedicines11082131

**Published:** 2023-07-28

**Authors:** José M. Inácio, Fernando Cristo, Miguel Pinheiro, Francisco Vasques-Nóvoa, Francisca Saraiva, Mafalda M. Nunes, Graça Rosas, Andreia Reis, Rita Coimbra, José Luís Oliveira, Gabriela Moura, Adelino Leite-Moreira, José António Belo

**Affiliations:** 1 Stem Cells and Development Laboratory, iNOVA4Health, NOVA Medical School|Faculdade de Ciências Médicas, Universidade NOVA de Lisboa, 1169-056 Lisbon, Portugal; jose.inacio@nms.unl.pt (J.M.I.); fcristo_10@hotmail.com (F.C.); mafalda.nunes@nms.unl.pt (M.M.N.); gracarosas@hotmail.com (G.R.); 2 Genome Medicine Lab, Department of Medical Sciences, Institute for Biomedicine—iBiMED, University of Aveiro, 3810-193 Aveiro, Portugal; monsantopinheiro@gmail.com (M.P.); areis@ua.pt (A.R.); racoimbra@aibili.pt (R.C.); gmoura@ua.pt (G.M.); 3 Cardiovascular R&D Centre—UnIC@RISE, Department of Surgery and Physiology, Faculty of Medicine, University of Porto, 1169-056 Porto, Portugal; fvasquesnovoa@med.up.pt (F.V.-N.); f.saraiva@med.up.pt (F.S.); amoreira@med.up.pt (A.L.-M.); 4 Institute of Electronics and Informatics Engineering of Aveiro (IEETA), University of Aveiro, 3810-193 Aveiro, Portugal; jlo@ua.pt

**Keywords:** heart failure, HFpEF, miRNA signature in HFpEF, miRNA–mRNA, intercellular communication

## Abstract

Heart failure with preserved ejection fraction (HFpEF) represents a global health challenge, with limited therapies proven to enhance patient outcomes. This makes the elucidation of disease mechanisms and the identification of novel potential therapeutic targets a priority. Here, we performed RNA sequencing on ventricular myocardial biopsies from patients with HFpEF, prospecting to discover distinctive transcriptomic signatures. A total of 306 differentially expressed mRNAs (DEG) and 152 differentially expressed microRNAs (DEM) were identified and enriched in several biological processes involved in HF. Moreover, by integrating mRNA and microRNA expression data, we identified five potentially novel miRNA–mRNA relationships in HFpEF: the upregulated hsa-miR-25-3p, hsa-miR-26a-5p, and has-miR4429, targeting HAPLN1; and NPPB mRNA, targeted by hsa-miR-26a-5p and miR-140-3p. Exploring the predicted miRNA–mRNA interactions experimentally, we demonstrated that overexpression of the distinct miRNAs leads to the downregulation of their target genes. Interestingly, we also observed that microRNA signatures display a higher discriminative power to distinguish HFpEF sub-groups over mRNA signatures. Our results offer new mechanistic clues, which can potentially translate into new HFpEF therapies.

## 1. Introduction

Heart failure (HF) affects close to 65 million people worldwide [1], being a leading cause of mortality, morbidity, healthcare resource consumption, and the main cause of hospitalization and disability among the elderly [1,2]. While the incidence of HF with reduced ejection fraction (HFrEF) has decreased in the last decade, HF with preserved EF (HFpEF) is growing with an incidence rate of 10% per decade and nowadays accounts for at least 50% of all HF cases [3,4], reflecting the ageing of the population and higher prevalence of cardiovascular comorbidities [3,4,5]. Importantly, both HFrEF and HFpEF present equally ominous prognoses, with a five-year mortality rate of 75% [6]. However, from a treatment perspective, HFpEF is notably more challenging, as traditional HF therapies targeting neurohumoral activation (e.g., beta-blockers and ACEi/ARBs) have not clearly shown clinical benefits in HFpEF.

Several pathophysiological mechanisms underlying HFpEF have been proposed. These include increased cardiomyocyte and extracellular stiffness, microvascular dysfunction, disturbances of cell communication, and gene expression programs involving multiple cell types (e.g., endothelial cells, fibroblasts, macrophages) [7,8,9,10]. Nevertheless, the precise molecular basis of these mechanisms in humans continues to be largely elusive. A substantial portion of our existing knowledge is reliant on animal models, which, although useful, are known to inadequately mimic the complexity and depth of human HFpEF, both in their clinical manifestations and molecular mechanics. This limitation becomes especially evident when considering non-coding RNA-related mechanisms, which are known to be poorly conserved across different species.

Here, we integrated left ventricular mRNA and microRNA expression data from patients with HFpEF and from a control group composed of aortic stenosis patients. We identified and experimentally validated five potentially relevant miRNA–mRNA interactions in HFpEF: the upregulated hsa-miR-25-3p, hsa-miR-26a-5p, and has-miR4429, targeting *HAPLN1*; and *NPPB* mRNA, targeted by hsa-miR-26a-5p and miR-140-3p. Moreover, we observed that microRNA signatures distinguished HFpEF sub-groups far better than mRNA signatures, most probably due to the noise introduced by comorbidities. These results might be considered in developing targeted therapies addressing HFpEF.

## 2. Materials and Methods

### 2.1. Patients

Cardiac biopsies were obtained from patients recruited to undergo cardiac surgery at Centro Hospitalar São João, as described previously [11]. All procedures were realized according to the Declaration of Helsinki and were approved by the local ethics committee and data protection authority (CES2006, 35/2017, and 1774/2017). All patients were assessed in terms of clinical characteristics, echocardiography, and blood sampling [11], and they were categorized into two groups: HFpEF according to ESC guidelines [2] and a non-HFpEF control group composed of aortic stenosis patients, from now on designated as “Control”.

### 2.2. Tissue Collection and RNA Extraction

Left ventricle tissue samples (~5 mg) were collected as described previously [11]. Total RNA was isolated from liquid nitrogen frozen samples using the Norgen Biotek kit (Norgen Biotek, Thorold, Canada), including a DNase treatment step. RNA concentration was measured with Nanodrop (Thermo Fisher Scientific, Waltham, MA, USA), and RNA quality was assessed with Agilent Fragment Analyzer (Agilent, Santa Clara, CA, USA).

### 2.3. RNA Sequencing

Only good quality total RNA (RIN > 6.5 and absorbance ratio 260/280 ~2) were used for library preparation. The preparation of the RNA seq and miR seq libraries and their deep sequencing were carried out by EMBL Genomics Core service facilities. Uni-directional deep sequencing of miR seq enabled ~10 million reads per library. A total of 60 million reads per library were obtained by total RNA seq.

### 2.4. mRNA Sequencing Analysis

For mRNA sequencing, the quality checking of the raw reads was performed with FASTQC v0.11.7 [12], and the parameters were adjusted to improve the quality of the reads with Trimmomatic v0.36 [13]. Then, the reads were mapped against the human reference genome (release-99/GRCh38, EBI) using STAR v2.7 [14]. Later, the mapped reads were tracked to protein-coding genes and counted using Stringtie v2.1.1 [15]. The genes were categorized into different biotypes and their distribution over the reference genome (EnsDb.Hsapiens.v86) using the NOISeq R package [16]. The raw counts were submitted to an in-house pipeline implemented using the DESeq2 R package [17]. Genes with no expression or those expressed in a single sample were excluded from further analysis. Standard DESeq2 normalization with RIN and human housekeeping genes (www.housekeeping.unicamp.br) (accessed on 19 June 2023) was carried out to reduce the statistical bias before differential gene expression analysis [18,19]. Principal component analysis (PCA) and unsupervised hierarchical clustering were performed with vst transformation (only for visualization purposes) using the prcomp and hclust R functions, respectively. Significant DEGs were identified by comparing samples of the HFpEF and control patient groups using a cut-off of false discovery rate (FDR) < 0.05.

### 2.5. miRNA Sequencing Analysis

For miRNA sequencing, after the reads’ quality control, an analysis was carried out using the QuickMIRSeq software [20] package, which uses Bowtie to perform the alignment and mapping against a database created with miRNA, hairpin, smallRNA, and mRNA sequences (with strand information), as concatenated from the latest versions of miRbase, GtRNAdb, and Ensembl. Subsequently, the program remaps the reads against the reference genome (UCSC database-version: hg38) considering mismatches–isomiRs to reduce the false hits. Afterward, to reduce background reads and improve miRNA detection, the program removes miRNA reads, which have an average number of counts of less than 2 per sample and are absent in more than 60% of the samples. Again, the raw counts were submitted to an in-house pipeline using the DESeq2 R package [17]. miRNAs with no expression or those expressed in a single sample were excluded from further analysis. Standard DESeq2 normalization was carried out including RIN values [19]. Principal component analysis (PCA) and unsupervised hierarchical clustering were performed with vst transformation (only for visualization purposes) using the prcomp and hclust R functions, respectively. Significant DEMs were identified by comparing samples of the HFpEF and control patient groups (FDR < 0.05).

### 2.6. miRNA–mRNA Interaction Analysis

Regarding the miRNA–mRNA interaction, first, normalized matrices were prepared from the mRNA and miRNA with the respective lists of DEGs and DEMs (FDR < 0.05). The miRComb R package [21] was used to find significant negative, positive, and two-sided correlations between the miRNA and mRNA normalized expression values. miRNA–mRNA pairs were considered significant when FDR < 0.05 and if their putative interaction information was present in at least one of the recommended databases (TargetScan_v6.2_20 and MicroCosm_v5_20), as recommended by mirComb authors.

### 2.7. Gene Enrichment, Network, and Pathways Analyses

Gene set enrichment analysis of gene ontology (GO) terms, with a focus on the biological process (BP), molecular function (MF), and cellular component (CC), was performed for DEGs using the clusterProfiler R package. For the pathway analysis, the Reactome web tool (https://reactome.org/) was used with mRNAseq and miRNAseq results. Additionally, network analyses and circos plots (miRComb package) were built to highlight the miRNA–mRNA interactions.

### 2.8. Cardiac Myocyte Culture

Primary human cardiac myocytes (HCMs) were commercially obtained from PromoCell, Heidelberg, Germany. HCMs were cultured in myocyte growth medium (PromoCell, Heidelberg, Germany) supplemented with 10% of Fetal Bovine Serum (Sigma-Aldrich, St. Louis, MI, USA) and incubated at 37 °C in a humidified atmosphere (95% air, 5% CO_2_). The medium was renewed daily, and cells were cultured in T-flasks and passaged to a 12-well plate using TrypLE Select (Thermo Fisher Scientific, Waltham, MA, USA) upon reaching 90% confluence.

### 2.9. miRNA Transfection

HCMs were plated on 12-well plates at a density of 15 × 10^4^ per well for 24 h before transfection in 1 mL of media without Pen/Strep. On the following day, upon reaching 90% confluence, cells were transfected with a mix of miRNA and Lipofectamine™ 3000 (Thermo Fisher Scientific, Waltham, MA, USA) at a final volume of 200 µL of Opti-MEM^®^ medium (Thermo Fisher Scientific, Waltham, MA, USA) without P/S and incubated for 24 h at 37 °C in a humidified atmosphere (95% air, 5% CO_2_). Several miRNAs were used for the transfection, including scramble as the negative control, as well as hsa-miR-25-3p, hsa-miR-26a-5p, hsa-miR-140-3p, and hsa-miR-4429 (Thermo Fisher Scientific, Waltham, MA, USA); their mature sequences are listed in Table 1. These miRNAs were used at a final concentration of 100 nM with five replicates each.

### 2.10. Isolation of Total RNA from Cell Cultures and Quantitative Real-Time PCR

After 24 h of transfection, the total RNA from HCMs subjected to a transfection protocol was extracted with the TRI Reagent^®^ (Sigma-Aldrich, St. Louis, MI, USA) and the Direct-zol^TM^ RNA MiniPrep Kit (Zymo Research, Irvine, CA, USA) according to the manufacturer’s instructions. The quantity and quality of RNA samples were assessed using a spectrophotometer (Nanodrop 2000, Thermo Fisher Scientific, Waltham, MA, USA). cDNA strands were synthesized through reverse transcription reaction using RevertAid Reverse Transcriptase, Oligo (dT) primers, RiboLock RNase Inhibitor, and dNTP (Thermo Fisher Scientific, Waltham, MA, USA). Amplification and fluorescent quantification were obtained from an ABI QuantStudio 5 Real-Time PCR System (Thermo Fisher Scientific, Waltham, MA, USA) using a SensiFAST SYBR Lo-ROX mix (BIOLINE, London, UK). RT-qPCR reactions were performed in triplicate. For normalization of the miRNA analysis, *GAPDH* and *β-ACTIN* were used as housekeeping genes. In addition, the relative quantification of target gene expression was performed using the ddCt method [22]. All primers used for amplification are listed in Table 2. Lastly, data were analyzed using GraphPad Prism version 8.0 for Mac (GraphPad Software). Statistical significance was determined using unpaired Student’s *t*-test. Differences were considered statistically significant when ** *p* < 0.01, *** *p* < 0.001.

## 3. Results

### 3.1. Patients

The clinical characteristics of the ten patients enrolled in this study are summarized in Table S1. They were divided into two groups, HFpEF (six patients) and Control (four patients), both with equal gender distribution (50% male and 50% female). The HFpEF group had a median age of 76 years, and the median age in the Control group was 58 years (*p* = 0.062). Hypertension (83% and 100%; *p* > 0.99) and diabetes (50% and 50%; *p* > 0.99) were present in the clinical history of HFpEF and Control groups, respectively.

### 3.2. mRNA Expression Profiles in HFpEF and Control LV Cardiac Biopsies

**Figure 1:**
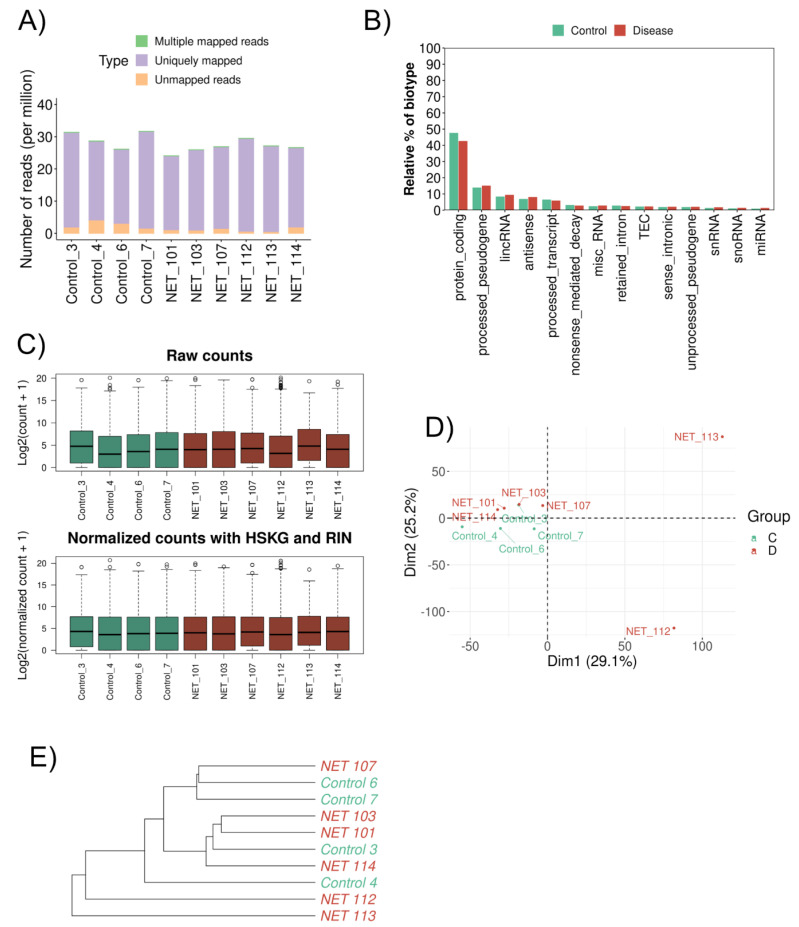
Gene expression differences between HFpEF and Control groups. (**A**) Bar plot showing the proportion of uniquely mapped, multiple mapped, and unmapped reads. The *x*-axis identifies the samples, and the *y*-axis shows the number of reads per million. (**B**) Bar plot comparing the relative abundance of each biotype (*y*-axis) in both sample types (*x*-axis). Green: Control; Red: HFpEF. (**C**) Distribution of read counts (log2) per sample (**A**) before normalization and (**B**) after normalization—HSKG normalization and RIN adjustment. Green: Control; Red: HFpEF. (**D**) Principal component analysis (PCA) of the mRNA from cardiac biopsy samples. Green: Control; Red: HFpEF. (**E**) Hierarchical clustering dendrogram using the Euclidean method for the 10 samples. Green: Control; Red: HFpEF.

The filtered RNA seq read set mapped an average of 26 million reads in all samples, varying from 23 to 30 million (Figure 1A). Regarding the relative abundance of the biotype (in percentage), the protein-coding genes were below 50%, the miRNA was around 1%, and the lincRNA and antisense were below 10% (Figure 1B). For further analysis, in addition to DESeq2 internal normalization, which corrects for library size and DNA composition bias, we used the RIN value and human housekeeping genes (HSKG) to complement the normalization of the samples (Figure 1C). Principal component analysis (PCA) distributed the patients (HFpEF and Control) homogeneously, separating only two HFpEF patients (Figure 1D). Unsupervised hierarchical cluster analysis also displayed the same similarity within the cohort (Figure 1E).

**Figure 2:**
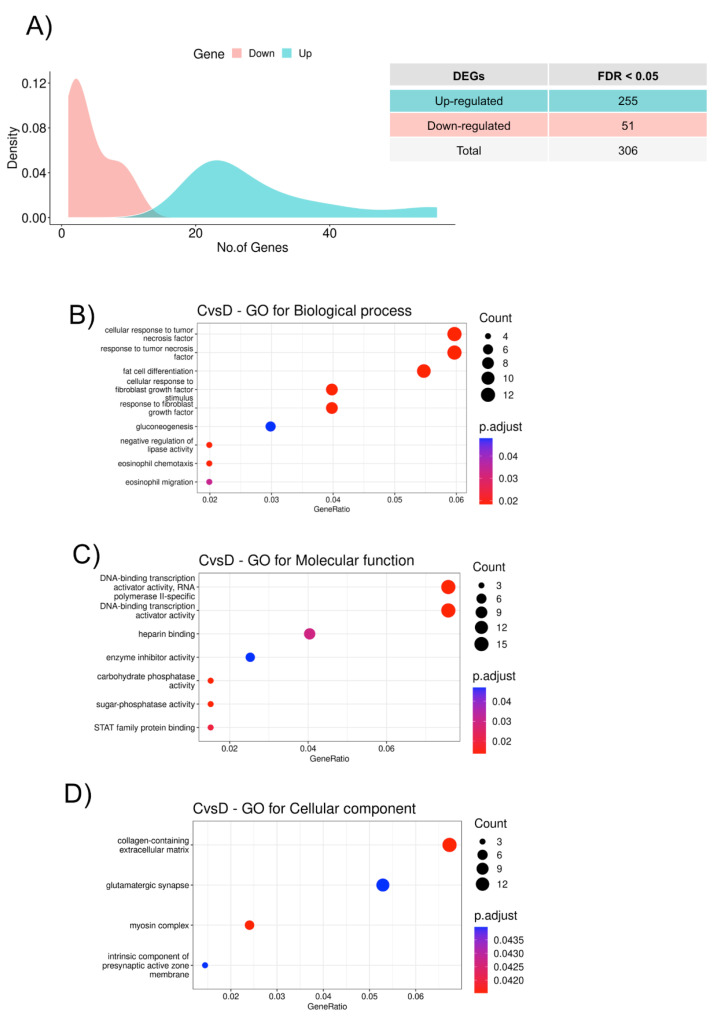
Functional classification of the DEGs. (**A**) Density plot showing the number of differentially expressed genes between HFpEF and Control groups with a false discovery rate (FDR) adjusted *p*-value < 0.05. The *x*-axis represents significantly dysregulated genes, and the *y*-axis shows the density of the log2 fold change. Downregulated genes are represented in red and upregulated genes in green. **(B**) GO biological process (BP) enrichment analysis, (**C**) GO molecular function (MF) enrichment analysis, and (**D**) GO cellular component (CC) enrichment analysis of the 306 significant DEGs from HFpEF vs. Control. The color of the dots indicates significance; the size of the dots indicates the gene count for each category; and the gene ratio indicates the enrichment level.

We identified a total of 306 differentially expressed mRNA genes (DEGs) (FDR < 0.05) between the HFpEF and Control groups: 255 upregulated and 51 downregulated (Figure 2A). These 306 genes were further analyzed in terms of the enrichment GO terms in order to obtain a functional insight into these genes. We observed that the mRNA DEGs in the HFpEF group are enriched regarding the cellular response to the tumor necrosis factor, fat cell differentiation, and cellular response to the fibroblast growth factor (Figure 2B); can function as transcription factors (Figure 2C); and are more related to the myosin complex and the extracellular matrix (Figure 2D). Overall, *LGI1*, *RYR1*, and *EFNB3* were the molecules, whose expression was particularly highlighted in the HFpEF group. Interestingly, *LGI1*, encoding Leucine-rich glioma-inactivated protein 1, *RYR1*, Ryanodine receptor 1, and the ephrin *EFNB3* are all molecules involved in the regulation of voltage-gated channels associated with HF.

### 3.3. miRNA Expression Signatures in LV Tissue in HFpEF Patients

**Figure 3:**
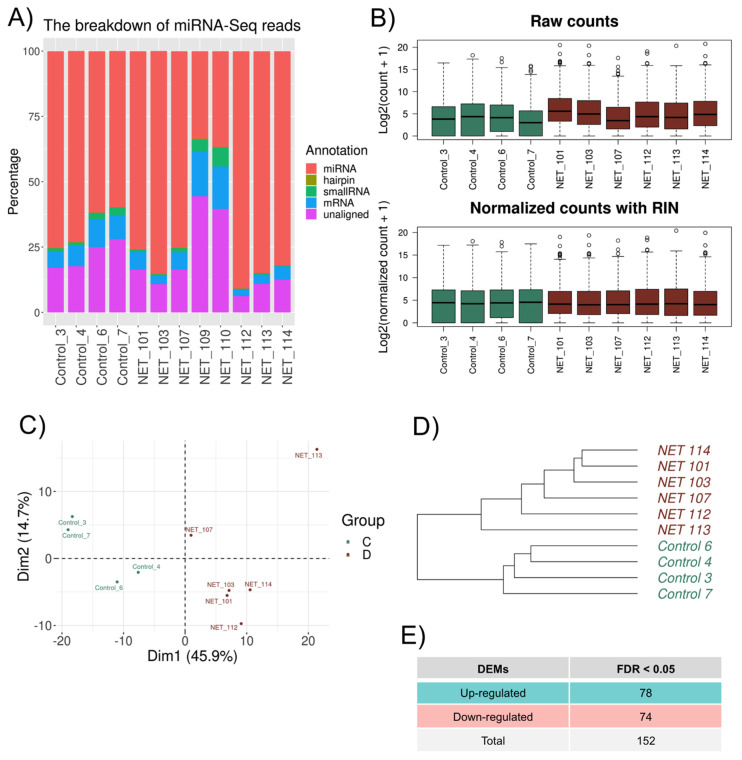
microRNA expression differences between HFpEF and Control groups. (**A**) Percentage of the reads mapped to different types of RNA and unaligned reads. (**B**) Distribution of the read counts (log2) per sample (**A**) before normalization and (**B**) after normalization—HSKG normalization and RIN adjustment. Green: Control; Red: HFpEF. (**C**) PCA score plot of the microRNA samples. Green and red colors represent the Control and HFpEF patients, respectively. (**D**) Hierarchical clustering dendrogram using the Euclidean method for the 10 microRNA samples. Green: Control; Red: HFpEF. (**E**) Summary of genes considered for microRNA–mRNA interaction analysis.

The results revealed an ordinary share of 25% of unaligned reads, and a more significant number of the mapped reads were miRNA (Figure 3A). Likewise, for mRNA normalization, the RIN information was used as a covariate, and a homogenized cluster for both the Control and HFpEF samples was obtained (Figure 3B).

The most abundant microRNAs were hsa-miR-944, hsa-miR-335-3p, hsa-miR-193b-3p, and hsa-miR-21-3p. PCA separated all expressed microRNA into two distinct groups matching the HFpEF and Control patients (Figure 3C). Moreover, the unsupervised hierarchical cluster analysis also identified the same two clusters within the cohort (Figure 3D). Regarding DEMs, 152 differentially expressed DEMs were identified among HFpEF and Control patients, with 78 DEMs upregulated and 74 downregulated among the HFpEF samples (Figure 3E).

### 3.4. miRNA–mRNA Interactions in HFpEF Patients

**Figure 4:**
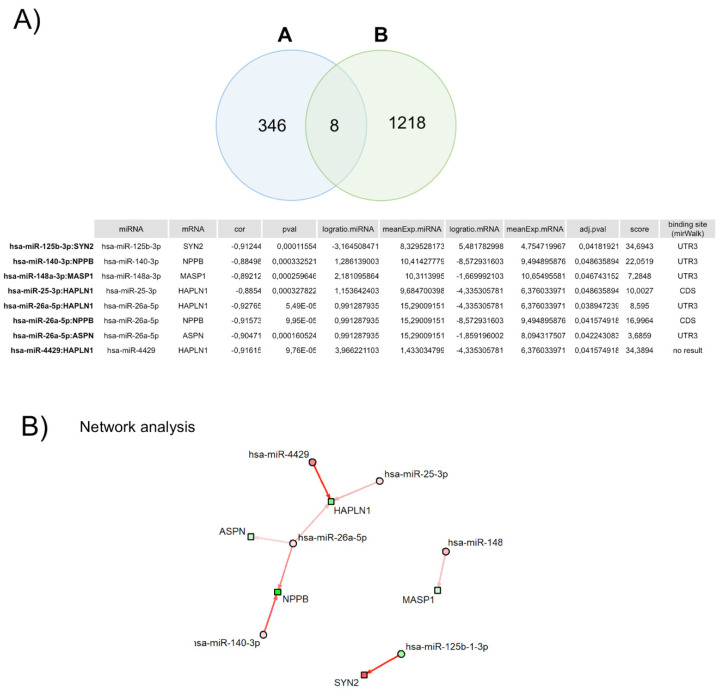
Correlation between each mRNA and corresponding miRNA(s). (**A**) Venn diagram of the number of miRNA–mRNA negative correlated pairs. (**A**) represents the number of pairs with correlation < 0 and padj < 0.05, and (**B**) represents the number of targets (miRNA–mRNA pairs) in public databases, which are present in our full dataset. (**B**) Network of miRNA–mRNA pairs with significant negative correlations (padj < 0.05). Circles represent miRNAs and squares mRNAs. Red fill means upregulated miRNA/mRNA, while green fill means downregulated miRNA/mRNA. Arrows indicate miRNA–mRNA pairs. Arrow color represents the score of the interaction; in this case, only weak-to-strong negative interactions (red arrows) are represented. Arrow width is proportional to the number of appearances in the database.

To identify miRNA–mRNA regulatory interactions in HFpEF, we used the previously determined 306 DEGs and 152 DEMs. The miRNA target prediction analysis identified a total of 46,512 possible significant interactions using the Pearson methodology. Using this criterion, correlations can be divided into positive, negative, or two-sided. Focusing our analysis on the negative correlations, we identified eight significant correlations (Figure 4A), one downregulated miRNA (miR-125b-1-3p), which leads to the upregulation of its target mRNA (positive logratio.mRNA), and five upregulated miRNA (miR-140-3p; miR-148a-3p; miR-25-3p; miR-26a-5p; and miR4429), inhibiting one or more target mRNAs (negative logratio.mRNA) (Figure 4B).

Further, we explored the predicted miRNA–mRNA interactions experimentally.

### 3.5. Transfection of miRNAs Confirms the in Silico Analysis

**Figure 5:**
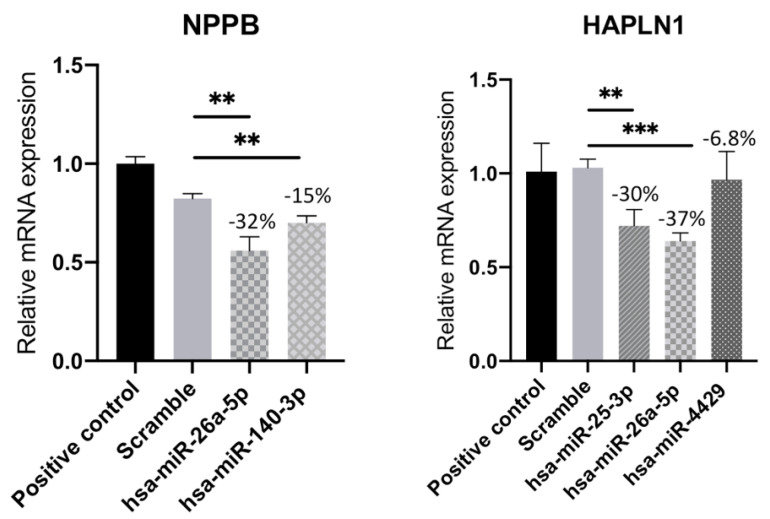
Relative mRNA expression of *HAPLN1* and *NPPB* genes in human cardiac myocyte line transfected with hsa-miR-25-3p, hsa-miR-26a-5p, hsa-miR-140-3p, or hsa-miR-4429. Results represent the mean ± SD of five replicates. Unpaired Student’s *t*-test was applied to compare the differences between conditions. Results were considered statistically significant when ** *p* < 0.01 and *** *p* < 0.001.

To validate the results obtained in silico, we proceeded to perform a functional analysis of the interaction in the HCMs cell line through miRNAs’ overexpression. In order to confirm the effect of miRNAs’ overexpression on the target genes, we performed real-time quantitative PCR. For this study, we focused on the genes *NPPB* and *HAPLN1*. The Natriuretic Peptide B (*NPPB*) gene is a member of the natriuretic peptide family and encodes a secreted protein, which functions as a cardiac hormone and has been widely used to diagnose HF [23]. The Hyaluronan and Proteoglycan Link Protein 1 (*HAPLN1*) gene has also been proposed to be involved in the process of HF for the first time [24]. Both genes have also been described to be expressed in the heart, more specifically in the cardiomyocytes [25], hence the use of the HCMs line for this functional analysis. The relationship between mRNA and miRNA sequencing performed previously predicted that the *NPPB* gene is a target of hsa-miR-26a-5p and hsa-miR-140-3p. The RT-qPCR analysis of this gene confirmed that transfection of HCMs with these miRNAs led to a downregulation of *NPPB* mRNA by 32 and 15%, respectively, compared with the negative control (scramble) (Figure 5). Regarding the *HAPLN1* gene, when the HCMs were transfected with three different miRNAs, we observed a downregulation of mRNA both in hsa-miR-25-3p and miR-26a-5p compared with the scramble, decreasing around 30 and 37%, respectively. On the other hand, there was a slight decrease of 7% when HCMs were transfected with hsa-miR-4429 (Figure 5). Overall, the RT-qPCR confirmed that overexpression of the several miRNAs mentioned above leads to inhibition and consequent downregulation of these two target genes, *NPPB* and *HAPLN1* (Figure 5).

## 4. Discussion

The substantial heterogeneity among HFpEF patients could be a significant factor contributing to the ongoing challenges in effectively treating the disease. At present, the therapeutic approach primarily involves the use of SGLT2 inhibitors, coupled with rigorous management of the risk factors, such as hypertension, diabetes, hyperlipidemia, and weight control [26]. Therefore, an improved understanding of the different HFpEF phenotypes might elucidate the underlying mechanism of the disease and help in the development of effective therapeutic approaches.

Aortic stenosis (AS) is a degenerative disease, which affects the aortic valve, resulting in chronic pressure overload on the left ventricle. While there may be some overlap with HFpEF in terms of age, prevalence of certain cardiovascular comorbidities, and a few cardiac structural features, AS is better understood from a mechanistic standpoint and is believed to primarily rely on pressure overload [27]. By studying the contrasting mechanisms at work in the myocardium between HFpEF and AS, as opposed to comparing them with young and healthy individuals, we gain valuable insights into the pathophysiology of HFpEF.

In this study, we sorted both differentially expressed genes (DEGs) and microRNA signatures from ventricular biopsies from HFpEF patients subjected to cardiac surgery. Importantly, these datasets allowed us to identify valuable microRNA–mRNA interactions, which one might consider apt for interventional targeting.

Of the 306 DEGs identified in HFpEF, the expression levels of *LGI1*, *RYR1*, and *EFNB3* were particularly increased in the HFpEF group. The *LGI1* gene encodes the Leucine-rich glioma-inactivated protein 1, which regulates voltage-gated potassium channels and is associated with sudden cardiac death [28,29]. Ryanodine receptor 1, *RYR1*, plays a role in regulating calcium channel opening and is behind the pathological mechanism involved in muscle weakness and impaired physical activity observed in patients with heart failure [30,31]. *EFNB3* has been associated with cardiovascular disease, mostly in blood pressure regulation [32]. At the molecular level, this ephrin is involved in myosin light chain kinase phosphorylation, which controls sensitivity to Ca^2+^ flux in vascular smooth muscle cells [32]. As a gene related to blood pressure regulation, such elevated expression of *EFNB3* in the HFpEF group is quite interesting, since the Control group patients were also hypertensive. Therefore, future investigation of the *EFNB3* function is necessary to further elucidate this ephrin’s role in HFpEF progression. Moreover, to our knowledge, none of the most significant DEGs identified in this study have been reported elsewhere, which demonstrates the heterogeneity among HFpEF patients [33,34,35]. Our PCA analysis also corroborates this observation, indicating that no consensus exists on the full myocardial gene expression signature in HFpEF. Nevertheless, *RYR* and *EFN* are, among others, the genes involved in calcium flux handling, which is important in diastole for removing Ca^2+^ from the cytosol, promoting cardiomyocyte relaxation [36].

For the differentially expressed microRNA (DEMs) identified, we found 152 significantly differentially expressed microRNA between the HFpEF and Control groups. The upregulated hsa-miR-21-3p and hsa-miR-193b-3p and the downregulated hsa-miR-8485 are microRNA associated with several mechanisms prompting heart failure [37,38,39]. The elevated expression of hsa-miR-21-3p in HFpEF patients was reported in a prior study [40] and was implicated in cardiac fibrosis in a HFpEF rat model [41]. The expression level of hsa-miR-193b-3p was found to be downregulated in the plasma of patients presenting HF [38]. However, in our analysis, this microRNA was found significantly overexpressed in the cardiac biopsies from HFpEF patients, suggesting diverse roles for hsa-miR-193b-3p in cardiac disease development. hsa-miR-8485 has been demonstrated to interact with the RNA-binding Protein TDP-43, whose aggregates are described as cardiac muscle degeneration markers [42]. However, the molecular mechanism of the downregulated has-miR-8485 in HFpEF remains to be determined. Interestingly, microRNA signatures have been demonstrated to distinguish HFpEF sub-groups with higher discriminative power [43]. Indeed, our PCA separated the expressed microRNA from the cardiac biopsies into two clear, distinct groups, matching HFpEF and Control patients. Therefore, microRNA signature may be usable as a potential therapeutic target enabling novel HFpEF therapies.

These microRNAs are part of an important class of RNAs, which regulate protein production post-transcriptionally, influencing the translation or the stability of the target gene mRNA. From a total of 46,512 miRNA–mRNA relationships, we found some significant miRNA–mRNA interactions already associated with cardiac diseases. For instance, inhibition of *KCNQ1OT1* by hsa-miR-140-3P exacerbates ischemia–reperfusion injury [44], and hsa-miR-140-3p inhibits cardiac hypertrophy through targeting *Gata4* [45]. Additionally, hsa-miR-148a-3p therapeutic delivery protected the mouse heart from pressure-overload-induced systolic dysfunction by preventing the transition of concentric hypertrophic remodeling, targeting the cytokine co-receptor glycoprotein 130 (gp130) connecting cardiomyocyte responsiveness to extracellular cytokines by modulating STAT3 signaling [46]. Importantly, we identified five potentially novel miRNA–mRNA relationships in HFpEF: the upregulated hsa-miR-25-3p, hsa-miR-26a-5p, and has-miR4429, targeting *HAPLN1*; and *NPPB* mRNA, targeted by hsa-miR-26a-5p and miR-140-3p. The hyaluronic acid (HA)-organizing factor HAPLN1 is required to produce hyaluronic-rich extracellular matrix needed for heart morphogenesis and injury-induced remodeling [47]. Moreover, the depletion of cells expressing *HAPLN1* impairs cardiomyocyte and cardiac fibroblast proliferation and inhibits heart regeneration or fibrotic remodeling [47]. *NPPB* is a cardiac gene encoding brain natriuretic peptide (BNP), which is expressed in both atrial and ventricular myocardium during heart development and strongly expressed in ventricular cardiomyocytes when the heart is overloaded [48]. Moreover, it has been shown that after heart failure, this cardioprotective hormone plays an important role in restoring the intravascular blood volume and vascular tone to physiologic levels [49]. Experimentally, we demonstrated that overexpression of hsa-miR-25-3p, hsa-miR-26a-5p, and has-miR4429, and hsa-miR-26a-5p and hsa-miR-140-3p drives the downregulation of the *HAPLN1* and *NPPB* genes, respectively. Despite the elevated levels of circulating B-type natriuretic peptide (BNP) observed in HFpEF patients, it is plausible to propose that this could interfere with the mechanotransduction mechanism, which regulates BNP production. This potential disruption can potentially decouple natriuretic peptide production from wall tension, thereby sustaining high filling pressures. The evaluation of these miRNA–mRNA relationships makes it clear that the development of therapeutic agents targeting these microRNA might be a promising strategy in HFpEF therapy. Therefore, the present study successfully identified the key miRNAs implicated in HFpEF patients with different comorbidities, aiming to provide new insight into the underlying pathophysiological mechanisms of HFpEF.

## Figures and Tables

**Table 1:** The mature sequences of miRNA.

miRNA	Mature Sequences
hsa-miR-25-3p	5′ CAUUGCACUUGUCUCGGUCUGA 3′
hsa-miR-26a-5p	5′ UUCAAGUAAUCCAGGAUAGGCU 3′
hsa-miR-140-3p	5′ UACCACAGGGUAGAACCACGG 3′
hsa-miR-4429	5′ AAAAGCUGGGCUGAGAGGCG 3′

**Table 2:** The sequences of primers used for quantitative real-time RT-PCR.

Target Gene	Sequence	Annealing Temperature (°C)
*NPPB*	Fwd 5′ CCCCGGTTCAGCCTCGGACT 3′ Rv 5′ ACGGATGCCCTCGGTGGCTA 3′	60
*HAPLN1*	Fwd 5′ GATACTGTTGTGGTAGCACTGG 3′ Rv 5′ TGCTGCGCCTCGTGAAAATTGAG 3′	59.8
*GAPDH*	Fwd 5′ GCTGGTAAAGTGGATATTGTTGCCAT 3′ Rv 5′ TGGAATCATATTGGAACATGTAAACC 3′	57.9
*β* *-ACTIN*	Fwd 5′ GCAAAGACCTGTACGCCAAC 3′ Rv 5′ AGTACTTGCGCTCAGGAGGA 3′	55

## Data Availability

The datasets presented in this study can be found in the following repository: https://www.ebi.ac.uk/ena (accessed on 19 June 2023); ID: PRJEB62450.

## Supplementary Materials

The following supporting information can be downloaded at: https://www.mdpi.com/article/10.3390/biomedicines11082131/s1, Table S1: Clinical characteristics of HFpEF and Control groups.
