# Supplementary material for: Myocardial RNA Sequencing Reveals New Potential Therapeutic Targets in Heart Failure with Preserved Ejection Fraction
Source: Biomedicines. 2023 Jul 28;11(8):2131. doi: 10.3390/biomedicines11082131 (PMC10452106; doi:10.3390/biomedicines11082131)
Supplement: Supplementary file 1 [file biomedicines-11-02131-s001.zip › biomedicines-2498725-supplementary.pdf]

Table S1 – Clinical characteristics of HFpEF and Control groups.

		Patients' characteristics				Comorbidities								
Patient	Group	Age	Sex	Weight (Kg)	BMI	AMI	ANG	Afib	PAD	CAD	HT	ANM	DM	COPD
NET_101	HFpEF	80	F	48	18,75	x	x			x	x	x		
NET_103	HFpEF	65	M	66	23,67		x			x	x			
NET_107	HFpEF	68	M	79	30,1	x				x	x			
NET_112	HFpEF	78	F	85	34,05		x				x	x	x	
NET_113	HFpEF	74	F	75	30,43		x					x	x	
NET_114	HFpEF	78	M	72	26,13	x			x	x	x		x	x
Ctrl_3	AS	58	F	109	45,36			x			x		x	
Ctrl_4	AS	50	F	116	38,75			x			x			
Ctrl_6	AS	58	M	75	26,89						x			
Ctrl_7	AS	79	M	67	25,52						x		x	

Legend: BMI-Body mass index; AMI – Acute myocardial infarction; ANG – Angina; Afib; Atrial fibrillation; AS – Aortic stenosis; PAD – Peripheral artery disease; CAD – Carotid artery disease; HT – Hypertension; ANM – Anemia; DM – Diabetes; COPD – Chronic obstructive pulmonary disease.
